# CartoMark: a benchmark dataset for map pattern recognition and map content retrieval with machine intelligence

**DOI:** 10.1038/s41597-024-04057-7

**Published:** 2024-11-08

**Authors:** Xiran Zhou, Yi Wen, Zhenfeng Shao, Wenwen Li, Kaiyuan Li, Honghao Li, Xiao Xie, Zhigang Yan

**Affiliations:** 1 Key Laboratory of Surveying and Mapping Science and Geospatial Information Technology of MNR, Chinese Academy of Surveying and Mapping, Beijing, 100036 China; 2 School of Environment and Spatial Informatics, China University of Mining and Technology, Xuzhou, 221116 China; 3 State Key Laboratory of Information Engineering in Surveying, Mapping and Remote Sensing, Wuhan University, Wuhan, 430079 China; 4 School of Geographical Sciences and Urban Planning, Arizona State University, Tempe, AZ 85287 USA; 5 Key Lab for Environmental Computation and Sustainability of Liaoning Province, Shenyang, 1100016 China; 6 Present Address: Key Laboratory of Geo-Information Engineering, Beijing, 100036 China

**Keywords:** Geography, Research data

## Abstract

Maps are fundamental medium to visualize and represent the real word in a simple and philosophical way. The emergence of the big data tide has made a proportion of maps generated from multiple sources, significantly enriching the dimensions and perspectives for understanding the characteristics of the real world. However, a majority of these map datasets remain undiscovered, unacquired and ineffectively used, which arises from the lack of numerous well-labelled benchmark datasets, which are of significance to implement the deep learning techniques into identifying complicated map content. To address this issue, we develop a large-scale benchmark dataset involving well-labelled datasets to employ the state-of-the-art machine intelligence technologies for map text annotation recognition, map scene classification, map super-resolution reconstruction, and map style transferring. Furthermore, these well-labelled datasets would facilitate map feature detection, map pattern recognition and map content retrieval. We hope our efforts would provide well-labelled data resources for advancing the ability to recognize and discover valuable map content.

## Background & Summary

**Fig. 1:**
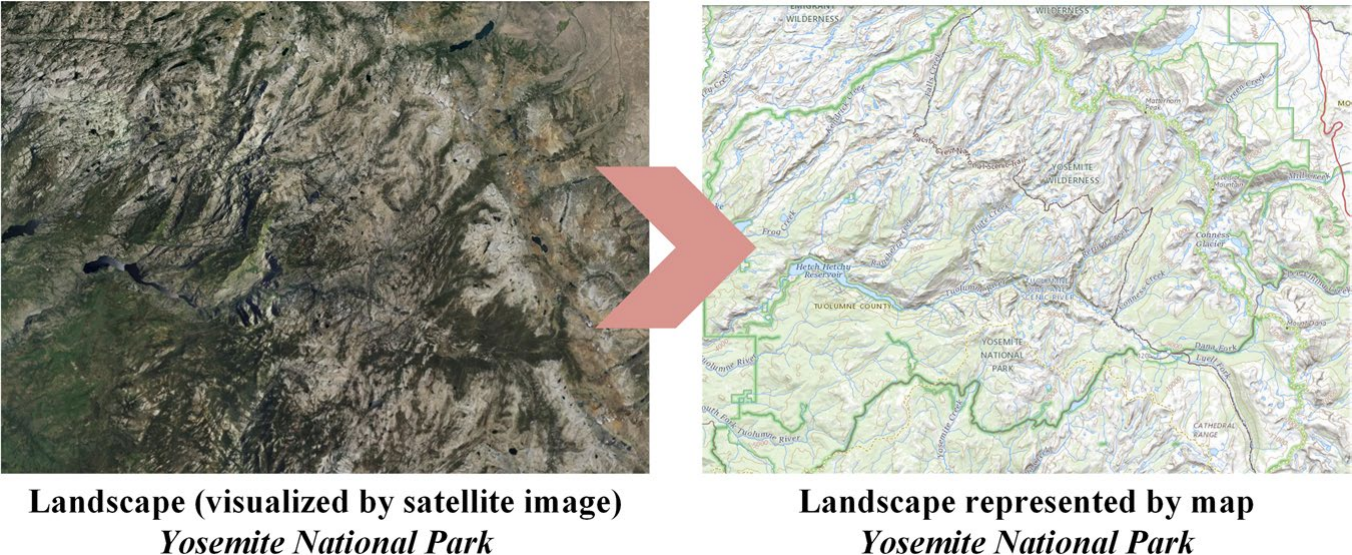
The visual representation of map on real landscape, in case of Yosemite National Park.

According to the definition of International Cartographic Association (ICA)^1^, a map is defined as “a symbolized representation of geographic reality, representing selected features or characteristics, resulting from the creative effort of its author’s execution of choices, and is designed for use when spatial relationships are of primary relevance.” In other words, maps serve as a medium to visualize the real word in a simple and philosophical way, and connect people to the reality through an inspired imagination^2^. As shown in Fig. 1, in comparison to the true representation of satellite image, maps are not necessarily a true representation of the real landscape, but rather an interpreted result. The information in a map reflects the map producer’s opinions and perspectives on a place, providing a unique viewpoint on the characteristics of this place. By comparing and contrasting the content from different maps on the same place, we can gain a deeper understanding of the varied perspectives and themes associated with that place, such as transportation, tourism, and civilization.

A variety of maps have been emerged as a powerful tool for communication and representation throughout human history, serving as one of the three common human being’ languages, alongside music and drawing^2^. Researches regarding cartography and its relationship to socio-economic development have reported that maps could represent various aspects of the natural and social worlds including scientific principles (e.g. portraying scientific principles), cultural and artistic expressions (recording the art and cultures), social opinions (depicting public opinions), historical events (visualizing historical maps status.), legal systems (displaying administrative boundaries), and military conflicts (illustrating the historical wars)^3–5^. Thus, recognizing and discovering the map information is of value to understand the natural and social characteristics of the place visualized by these maps, and enhance the capability of geospatial analysis on natural landscape and socio-economic development.

The emergence of big data tide has revolutionized the paradigm of cartography and map analysis approaches, as massive maps are now available to be created, edited, published and shared to represent the producers’ personal viewpoints and subjective understandings. This has led to an explosion in the volume and variety of geospatial information available from massive maps generated by different countries, institutes and people are presented through various platforms including earth observation system, information cyberinfrastructures, and big data techniques^2,6^. Thus, classical non-content-based approaches such as metadata-based map analysis^7^ and file name-based map analysis^8^ might not be effective in recognizing the content in the maps accessed from massive sources. This is because the metadata and title of these maps often subjective and may vary making it difficult to accurately identify and retrieve specific maps^9^. Expert-based intelligent systems have been developed for map content analysis^10^. However, these labor-driven machine-person interactions are time-consuming and might be insufficient for addressing the diverse map content available from massive sources^11^. Other approaches including image morphology^12^, object-based feature detection^13^, and object-based image analysis^14–16^ always requires a manually-defined threshold, which are labor-intensive and inaccurate.

**Fig. 2:**
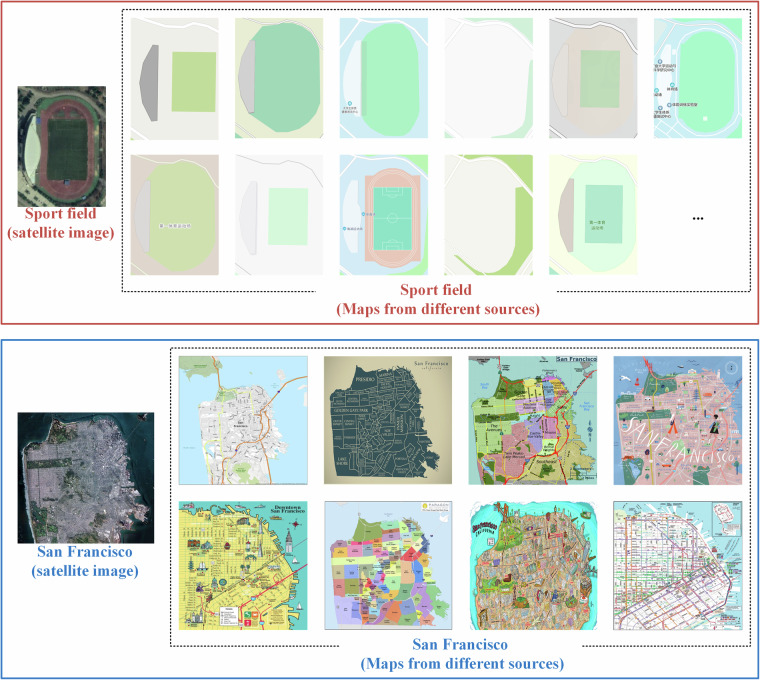
Comparison of maps from different sources: in case of San Francisco and sport field.

Deep learning techniques have garnered a significant attention in the communities of cartography and geospatial information science because of its powerful capability of feature learning. In previous decade, the-state-of-the-art machine learning and deep learning approaches have been employed to effectively extract map features and discover the geospatial information from standard maps being generated by professional mapping principles^17–19^. The deep learning approaches includes convolutional neural networks (CNNs) and vision transformer (ViT) regarding map scene classification^20^, multi-task classification^21^, map feature detection^22^, and map feature segmentation^19,22^. However, these approaches have been reported inefficient for addressing the content from volunteered maps, which are generated from diverse ways that fail to follow cartographical principles. The styles, symbols, scenes, and other map features in these maps can vary significantly, posing a challenge for developing a benchmark dataset that includes all types of map samples in sufficient numbers^2,6,23^. This makes it difficult for deep learning approaches to learn salient features from the map content. Figure 2 illustrates the varied representation of maps: the same sport field is visualized similar under different satellite images, but differently by different maps. This highlights the challenge of map recognition, which has to consider the difference of different maps. As a result, the researches of cartography and location-based services have found that a majority of map dataset have been unused and underutilized^24,25^, leading to the waste of plentiful information contained within these maps. Moreover, the map data used in many applications may not be entirely suitable for the authentic demands of these applications, resulting in the squandering of cartographical data processing and analysis^6,26^

Above of all, deep learning approaches always heavily rely on numerous well-labelled datasets. The absence of a benchmark dataset for map content hinders the implementation of deep learning techniques in identifying complex map content. Given the potentials of maps to enhance a variety of applications in terms of cultural narratives, navigations, location-based services, etc., the gap between sufficient map data and unproductive map utilization pose an urgent demand: developing a large-scale benchmark dataset that facilitate the state-of-the-art machine intelligence technologies to accurately detect map features, recognize map patterns and conduct map content retrieval. To address this need, we present the details of a large-scale benchmark dataset called *CartoMark*. Our goal is to provide a comprehensive dataset that enables researchers and practitioners to utilize cutting-edge machine intelligence techniques and evaluate their proposed approaches for map pattern recognition and map retrieval. By developing this dataset, we aim to facilitate the development of more accurate and efficient machine intelligence algorithms for map analysis, ultimately enhancing the utility of maps in various applications.

## Methods

### Data sources

Maps in *CartoMark* are accessed from three main types of source: Internet, data cyberinfrastructure/repositories, and social media. The maps on the Internet are accessed by commonly-used search engines including Google image search, Bing image search, and Baidu image search. The data cyberinfrastructure/repositories for collecting maps include USGS Earth Explorer, USGS Historical Topographic Map Explorer, and Tianditu Map. A small proportion of maps are accessed from the third data source—social media including Facebook, Twitter, Instagram and TikTok.

### Architecture of CartoMark

**Fig. 3:**
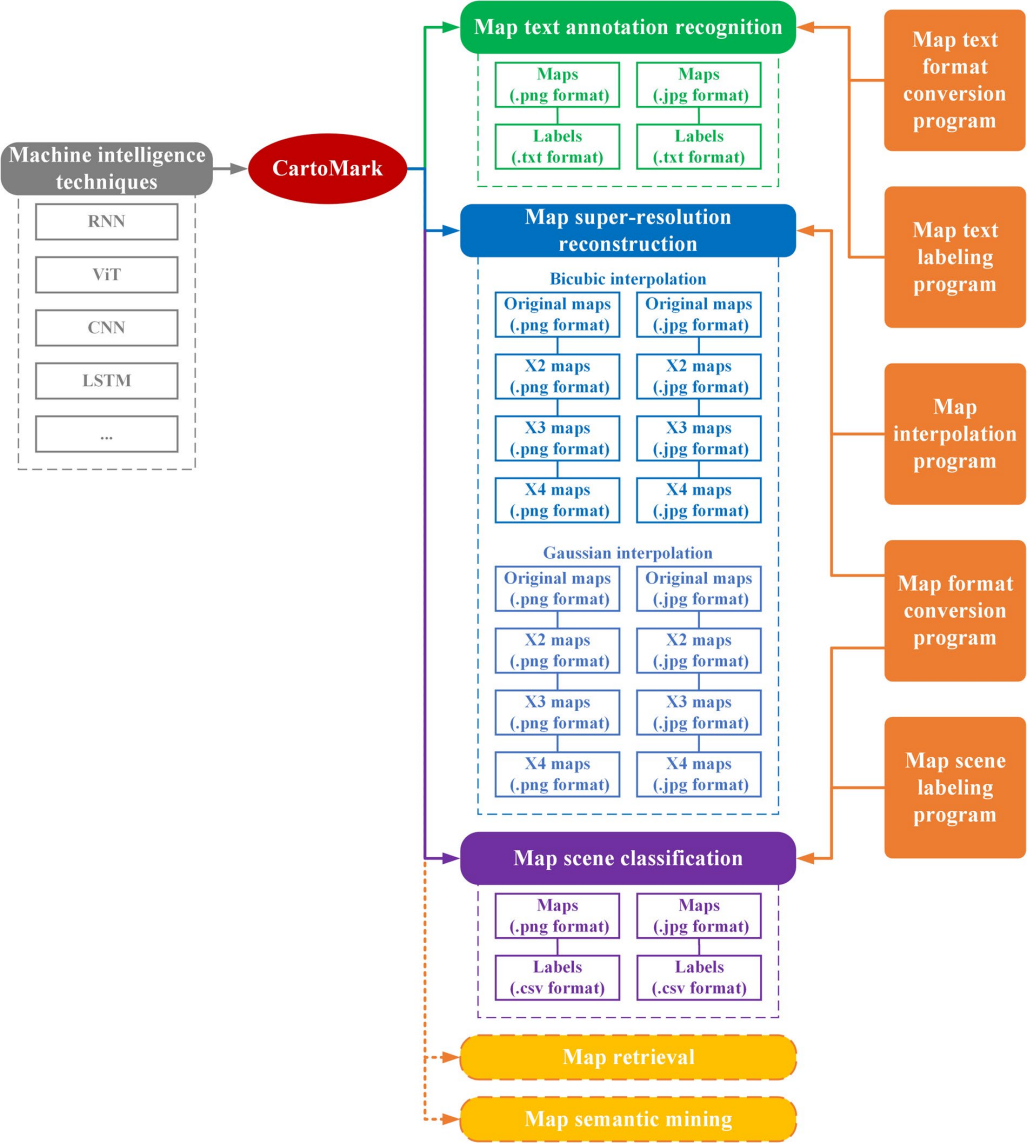
Architecture of CartoMark.

Figure 3 illustrates the architecture of *CartoMark*, which supports four tasks of cartographical pattern recognition: map text annotation recognition, map super-resolution reconstruction, map scene classification, and map style transferring. Moreover, all datasets compatible with the-state-of-the-art machine intelligence techniques.

- Map text annotation recognition aims to detecting and recognizing the text characters in maps. Its repository includes two data groups, with identical map files i in two formats: JPG and PNG. Each group provides map files and corresponding label files.
- Map super-resolution reconstruction focuses on generating the higher resolution maps from the low-resolution original maps. Its repository includes two groups of map files, identical in content but different in format: JPG and PNG. Each group provides map files alone.
- Map scene classification concentrates on classifying maps into various categories based on their content. Its repository includes two data groups, with identical map files in different formats: JPG and PNG. Each group provides map files and corresponding label file.
- Map style transferring focuses on transferring the original maps into specific styles while maintaining the initial content. Its repository includes two groups of map files, identical in content but different in format: JPG and PNG. Each group provides map files alone.
  To accommodate the input data requirement of various the state-of-the-art machine intelligence techniques, we provide two commonly-used map formats (JPG and PNG). Additionally, we offer a format conversion tool for users to convert the current formats into bmp. and tif. without any additional operations.
  Besides datasets, we also provide three python-encoded programs (or tools) for users to process the map samples, which includes Image format conversion, Text line drawing, and Text annotation formation conversion.
- *Image format conversion*: a tool to convert the available map format into other main formats.
- *Text line drawing*: a tool for visualizing the bounding box that covers a map text.
- *Text annotation format conversion*: a tool to transform the data structure of.txt files into other data structures required by various deep larnin techniques regarding optical character recognition (OCR).

### Taxonomy and folksonomy integrated map harvesting

1. **Map-dataset harvesting**
  As mentioned above, maps from ubiquitous sources, which refers to the sources that maps are generated are distributed everywhere on the Internet, are always generated by different ways, thereby requiring a diverse approach to harvest original map dataset. Maps generated by professional cartographical rules can be assessed using standardized cartographical taxonomies. However, for the maps generated by volunteered ways that holds the similar content, it might include different names (tags), which belongs to folksonomy in the information retrieval. Thus, we combined standardized cartographical taxonomies for professionally-generated maps with folksonomies from volunteered maps to create keywords for harvesting or retrieving the original map dataset. The taxonomy and folksonomy integrated keywords are listed in Table 1.

**Table 1:** List of the taxonomy and folksonomy integrated keywords.

Keyword categories	Keyword individuals
City	Beijing, Shanghai, Guangzhou, Xi’an, Shenzhen, Hangzhou, Nanjing, New York, Washington DC, Los Angeles, San Francisco, Seattle, Chicago, Philophobia, London, Manchester, Paris, Madrid, Barcelona, Berlin, Frankfurt, Munich, Hamburg, Milan, Turin, Roma, Amsterdam, Moscow, Istanbul, Vienna, Prague, Stockholm
Continent	North American, African, Asian, Europe, South American
Space	three-dimensional, 3D,virtual, bird-eye
Nature	hydrology, water net, topographical, terrain, digital elevation model, TIN, digital surface model
Social-economy	indoor, shopping mall, airport, museum, cinema, travel, tourism, urban, traffic, street, land classification
Others	comic, drawing,
  Based on these keywords, we employed a search strategy that combined “keyword individual + map” to retrieve and collect map files from various sources, including commonly-used search engines, data cyberinfrastructure/repositories, and social media crawlers. For example, we used “San Francisco map”, “indoor map”, “airport map”, etc. to retrieve and collect all relevant map datasets. As mentioned above, the commonly-used search engines used include Google Image Search, Amazon Image Search, Yahoo Image Search, Bing Image Search, and Baidu Image Search. Specifically, all map datasets accessed from these commonly-used search engines are checked with consent. If a map dataset could be accessed but downloaded, we would remove this map datasets.
  Data cyberinfrastructure/repositories including USGS Earth Explorer, USGS Historical Topographic Map Explorer, and Tianditu Map were also used, although the number of maps retrieved from these sources was limited due to their similar map styles and configurations. Social media crawlers were used to retrieve maps from platforms such as Facebook, Twitter, Instagram, and TikTok, but the amount of maps obtained from these sources was also limited due to the majority of social media platforms not being publicly accessible.
  In total, around 20,000 maps were collected from the three sources using the map-dataset harvesting approach, and were stored in the original maps. The majority of the maps were obtained from web search engines.
2. **Map-dataset cleaning and map filtering**

Since a majority of retrieved original map datasets were generated by unprofessional ways, they may lack the necessary accuracy and clarity required for effective map pattern recognition. To address this issue, we propose an integrated strategy to filter and clean the original map datasets being unqualified to meet the required standards for use in benchmark datasets.. The workflow of this strategy includes five steps: image size filtering, image format unifying, image color and texture filtering, image noise removing and image content checking.

1. Map resizing.

- We set the minimal image size as 256*256, and removed all maps that smaller than this minimal size in horizontal or vertical dimension. The minimal size was determine based on the requirement of input data dimension of the state-of-the-art deep learning approaches.
- For maps between 256*256 to 512*512 pixel, we cropped the central part of this map as the map sample in CartoMark.
- For maps between 512*512 to 1024*1024 pixels, we would randomly partition this original map into two sub-parts as the map samples.
- For maps larger than 1024*1024 pixels, we would randomly partition this original map into four sub-parts as the map samples. The cropping and partitioning were conducted according to the following rules.

Assuming the dimension of an original map dataset as (*x*, *y*), for the dimension proposal $(d,d=\,\max (x,y))$,

If $d=\in (256,512)$, we cropped the central part of this map as the map sample, and the area of the cropped map is $(\frac{1}{4}x:\frac{3}{4}x,\frac{1}{4}y:\frac{3}{4}y)$.

If $d=\in (512,1024)$, we randomly partitioned the map into two parts, and then selected the central part of each part as the original map samples.

If $d=\in (1024,+\infty )$, we randomly partitioned the map into four parts, and then selected the central part of each part as the original map samples.

Because a proportion of volunteered maps were limited in size image resizing can filter a great amount of original map dataset. In practice, we generally removed around 8000 map datasets in this step.

2. Map reformatting. Besides size issue, a proportion of the maps were not created in a machine-readable format, and we removed these unreadable maps by commonly-used data processing tools including OPENCV, Adobe Photoshop, and ArcGIS. The selected format included JPG, JPEG, PNG, BMP, TIFF, GIF, AI, CDR, EPS, SVG, and PSD. Moreover, for the dynamic maps generated by TIFF, we splitted them into multiple frames, and randomly selected one frame as the map sample. Finally, we converted all selected map samples into two format groups: JPG and PNG.
3. Map color and texture filtering. Some map datasets might have a visually-incorrect colors and textures due to a variety of reasons, such as format conversion, data editing, data quality, etc. Since the incorrect color and texture of these maps were not suitable for visual cognition and machine intelligence techniques, we manually removed these map datasets.
4. Image noise filtering. Removing various types of noises in these maps generated by unprofessional ways would always be challenging with automatic methods. However, the capability of addressing noises is critical to the performance of the-state-of-the-art approaches for map pattern recognition. Thus, we manually checked all maps by visual interpretation, and removed the original map datasets that had significant noises affecting the representation of map content.
5. Image content filtering. It is widely acknowledged that information from the Internet and cyberspace might contain illegal or immoral clues. As the benchmark dataset would be published and shared by the global users, we also manually checked all maps by visual interpretation and removed any maps that might include inappropriate or immoral clues.

By implementing this integrated strategy, we can ensure that the original map datasets are of high quality and meet the necessary standards for use in benchmark datasets. After cleaning and filtering, the total number of the map samples in CattoMark are listed in Table 2.

**Table 2:** Total number of map samples in CartoMark.

Map category	Interpolation methods	Map format	Total map number	Total label number
Map Scene Classification	—	.jpg/.png	10377	10377
Map Text Annotation	—	.jpg/.png	455	455
Map Super-resolution	Bicubic	.jpg/.png	1100	1100
	Gaussian	.jpg/.png	1100	1100

### Map labelling

**Fig. 4:**
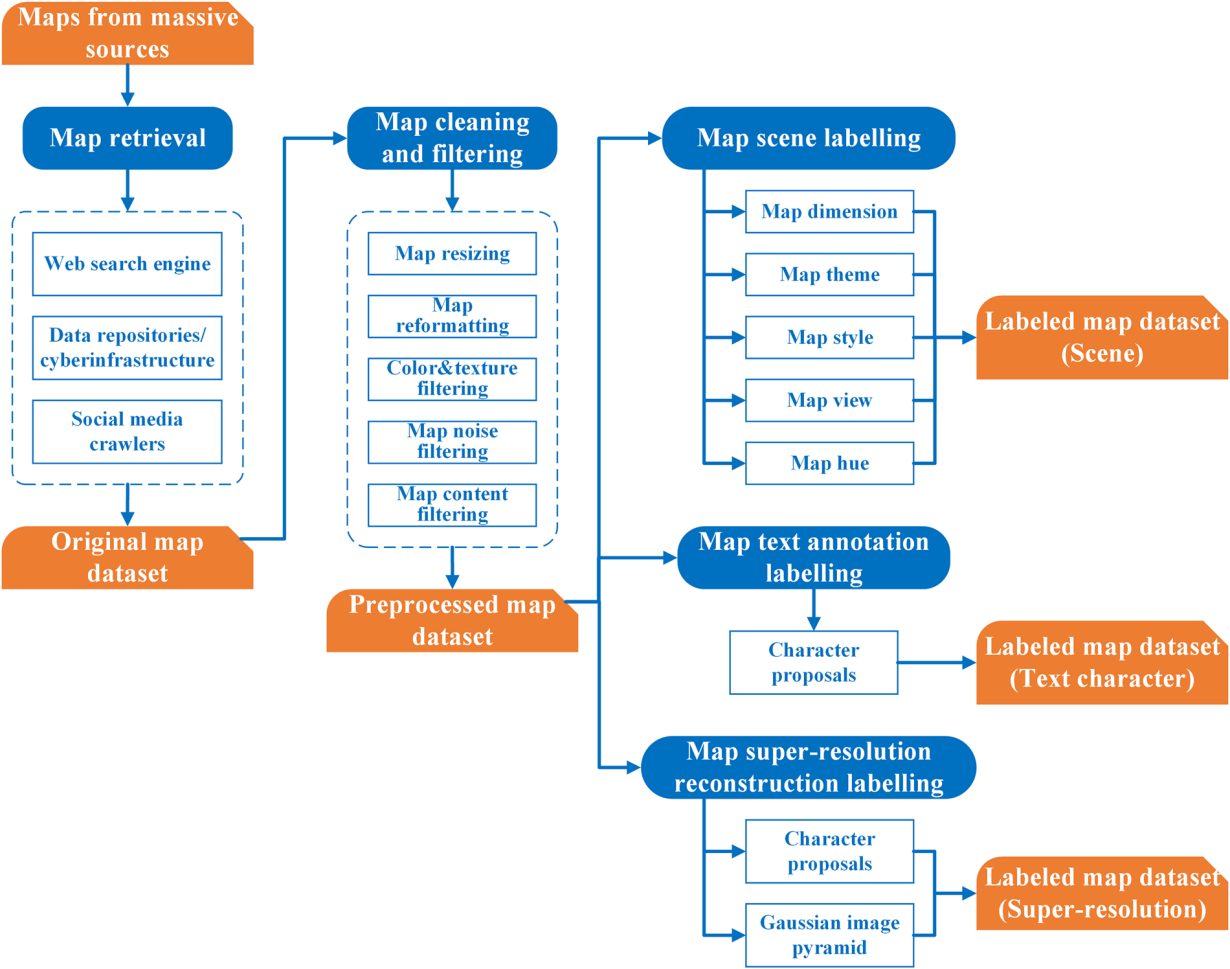
Workflow of map labelling.

The map labelling process is depicted in Fig. 4. For each collected map sample, we first assigned a scene category label, and then generated the labelled map dataset regarding map scene. Next, we assigned a character proposal for each text unit, and then generated the labelled map dataset regarding text character. Finally, we respectively created two groups of low-resolution maps by bicubic interpolation and Gaussian filtering, and then generated the labelled map dataset regarding super-resolution reconstruction.

(1) **Map scene labelling**

**Fig. 5:**
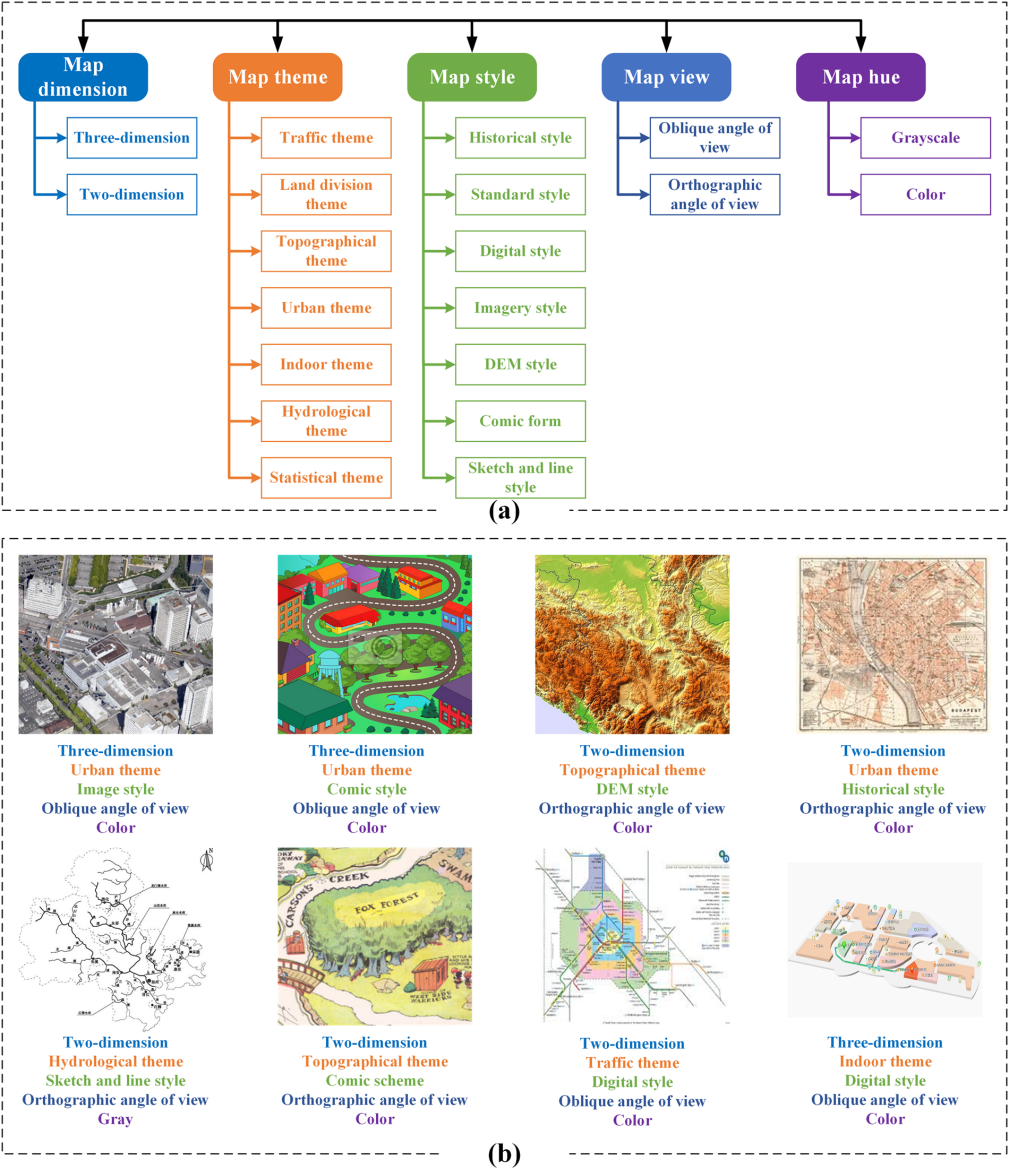
Hierarchical labelling system of CartoMark. (**a**) Hierarchical labelling system, and (**b**) the example.

Based on the taxonomies defined by the cartographical classification systems and standards involving Chinese topographical mapping standard (GB/T 16820-2009: https://openstd.samr.gov.cn/bzgk/gb/newGbInfo?hcno=AADA46D2F301C30AF9103A6789C40089), USGS national maps (https://store.usgs.gov/) and land classification legend (https://www.usgs.gov/media/images/land-cover-class-legend), United Nations Maps&Geoservices (https://www.un.org/geospatial/mapsgeo/thematic), and European landscape classification (https://ec.europa.eu/eurostat/cache/metadata/en/lan_esms.htm), we designed a multi-dimensional hierarchical labelling system as shown in Fig. 5a.

The labelling system includes five dimensions of hierarchies: map dimension, map theme, map scheme, map view and map hue. Moreover, each dimension includes various sub-categories. The details of these five dimensions of hierarchies are listed in Table 3.

**Table 3:** The details of five dimensions of hierarchies.

Map dimension	Two-dimension map	A map that represents the landscape in a flat, two-dimensional space.
	Three-dimension map	A map that represents the landscape in a three-dimensional space.
Map theme	Traffic theme	A map that mainly represents the geospatial information regarding traffic, such as roads, highways, intersections, and other transportation infrastructure.
	Land division theme	A map that mainly represents the geospatial information regarding separate land parcels, such as land classification map, administrative boundary map, population map, etc.
	Topographical theme	A map that mainly represents the geospatial information regarding relief features and landform, such as contour lines.
	Urban theme	A map that mainly represents the geospatial information regarding urban area. This type of map it includes other features besides traffic ones.
	Indoor theme	A map that represents the geospatial information regarding indoor features, such as shipping mall map, airport map.
	Hydrological theme	A map that represents the geospatial information regarding water network and hydrological features.
	Statistical theme	A map that represents the gradual changes of geospatial features over the global map space, such as temperature map, air quality map, quantitative pollution map.
Map style	Historical style	A map being generated by ancient mapping methods. This type of map are always historical maps, which are printed on a paper.
	Standard style	A map being generated by professional mapping ways, such as topographical maps created by trained cartographers.
	Digital style	A map being generated by volunteered mapping ways, such as openstreet map.
	Imagery style	A map being generated by image.
	DEM style	A map being generated by digital elevation models, providing terrain features and details.
	Comic style	A map being generated by comic mapping ways.
	Sketch and line style	A map being generated by sketch mapping ways, using simple lines and points.
Map view	Oblique angle of view	A map that represents the content with oblique angle, providing a bird’s eye view.
	Orthographic angle of view	A map that represents the content with orthographic angle.
Map hue	Grayscale	A map that represents the content by grayscale color bar.
	Color	A map that represents the content by color bar.

So, each map sample would be labelled as five categories. Moreover, any individuals in each hierarchy are independent and exclusive. For example, a map could be labelled as: *{two-dimension, traffic theme, digital scheme, orthographical angle of view, color}*, but not *{two-dimension, traffic theme, topographical theme, digital scheme, orthographical angle of view, color}*, as traffic theme and topographical theme belong to the same hierarchy, and are mutually exclusive. The examples are shown in Fig. 5b.

(2) **Map labelling for text annotation**

The workflow of map labelling for text annotation includes two sequential steps: selecting map text annotation proposal and labelling text position. We created the map text annotation proposals based on the collected map samples, adhering to the following criteria::

- each proposal has distinct styles (e.g. variations in character form, glyph, color, and other features);
- each proposal has different character arrangements (e.g. curved or rotated characters, etc.);
- each proposal has varied backgrounds (e.g. noises, other map features that overlay characters).

**Fig. 6:**
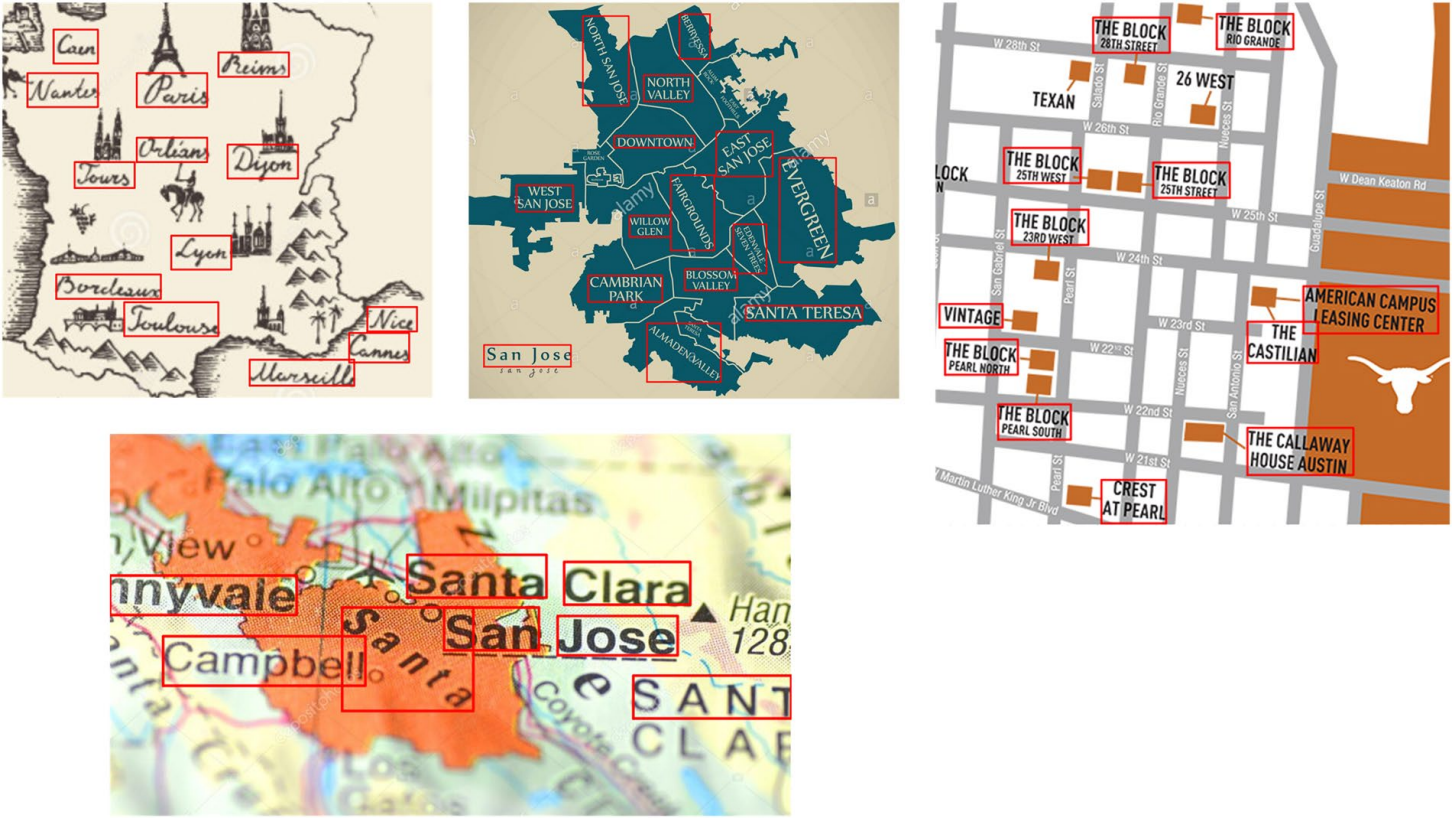
The example of map labelling for text annotation.

Then, we use a state-of-the-art labelling tool called PaddleOCR to label the position of each text in a map. PaddleOCR can be accessed by this link: https://github.com/PaddlePaddle/PaddleOCR (accessed date: 04-25-2024). We draw a four-side polygon (rectangle or square) minimal bounding box for every map text in each map, and recorded the coordinates of four corners of the bounding box. The coordinate refers to the position of pixel that located at the corner of this minimal bounding box. Figure 6 displays the selected result of map labelling for text annotation, the product of which includes the map file and the red bounding box that represents the positions of each text.

(3) **Map labelling for super-resolution reconstruction**

The workflow of map labelling for super-resolution reconstruction includes map proposal selection and map interpolation. We selected the collected map samples as map proposals. Then, for each map proposal, we created three blurred maps (or low-resolution maps) by cubic spline interpolation and Gaussian filtering, respectively, with 2X, 3X and 4X scales. The details of cubic spline interpolation can be read in Reference^27^, and the function of Gaussian filtering is expressed as follows,

1 $$G(x,y)=\frac{1}{2\pi {\sigma }^{2}}{e}^{-({x}^{2}+{y}^{2})/2\pi {\sigma }^{2}}$$

where (*x*,*y*) refers to the position of a pixel, *σ* refers to the variance.

**Fig. 7:**
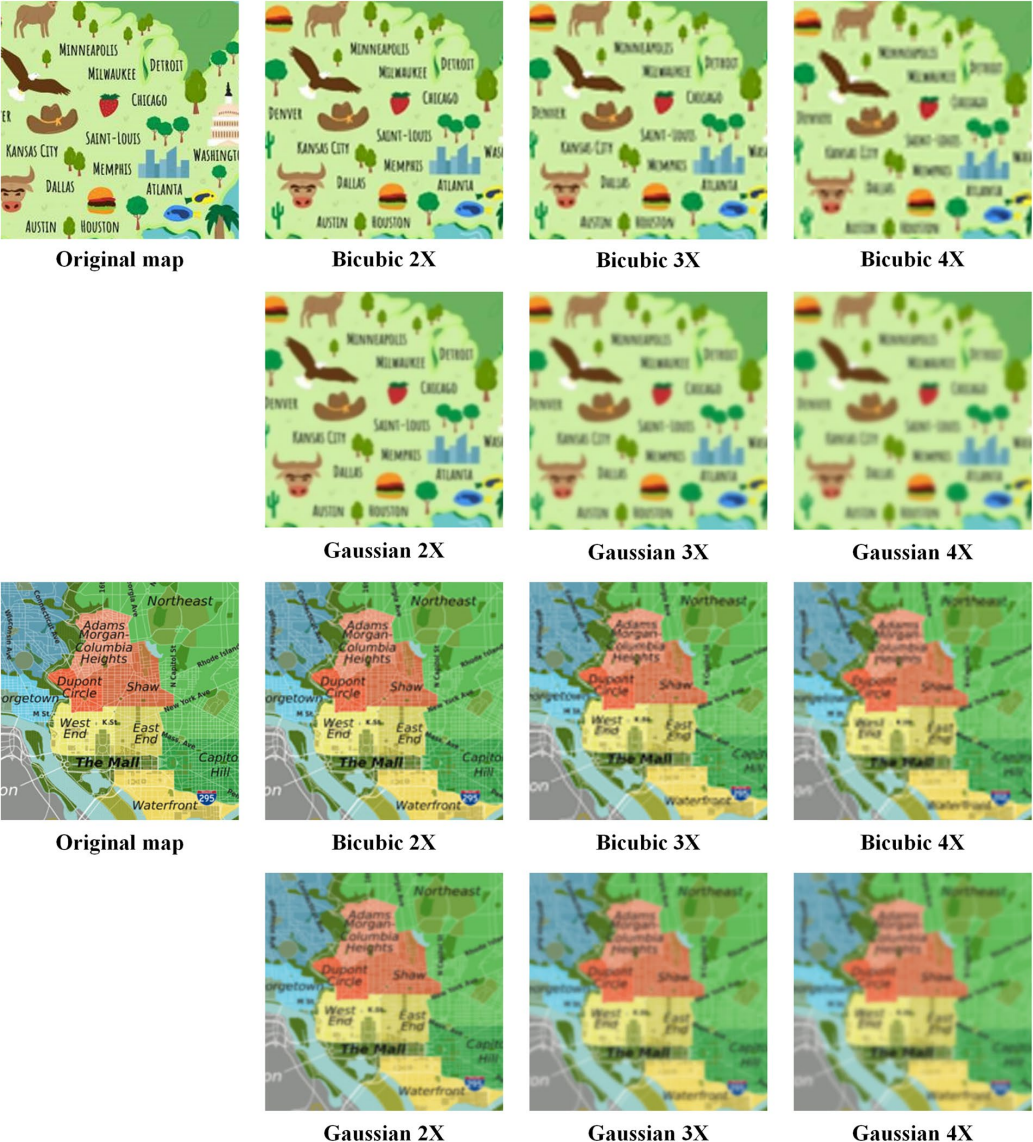
The example of map labelling for super-resolution reconstruction.

Figure 7 illustrates the selected results of the map labelling for super-resolution reconstruction process. The resulting product comprises the original map file, a set of maps containing the 2X, 3X,and 4X map files generated by Gaussian filtering and another set of files containing the 2X, 3X,and 4X map files generated by cubic spline interpolation.

(4) **Map labelling for style transferring**

The labeling for map style transferring is used for transforming the original style of a map into other styles by the state-of-the-art deep learning techniques. The workflow of map labelling for style transferring includes style proposal selection and map style collection building. We used the collected map samples for map scene classification as style proposals, and organized them into map style collection.

## Data Records

The benchmark dataset can be accessed by the following repositories:

- Harvard Dataverse^28^: https://dataverse.harvard.edu/dataset.xhtml?persistentId=doi:10.7910/DVN/ZBXJD5.
- Github: https://github.com/xrzhou/CartoMark.

As shown in Fig. 2, the datasets in CartoMark supports three tasks: map text annotation recognition, map scene classification, map super-resolution reconstruction, and map style transferring. The data modal and data format for each task are listed in Table 4.

**Table 4:** Data modal and data format of CartoMark.

Category	Data modal	Data format
Map text annotation recognition	Image	.JPG .PNG
	Text	.TXT
Map scene classification	Image	.JPG .PNG
	Text	.CSV
Map super-resolution reconstruction	Image	.JPG .PNG
Map style transferring	Image	.JPG .PNG

The datasets of map text annotation recognition involve two modalities: imagery and text. The imagery modal dataset includes map images in two commonly-used formats: .JPG, and .PNG. The text modal dataset records the coordinates of each text proposal and is available in .TXT format.

The datasets of map scene classification also involve two modalities: imagery and text. The imagery modal dataset consists of two aforementioned formats: .JPG and .PNG. The text modal dataset records the category of each map image and is available in .TXT format.

The datasets of map super-resolution reconstruction involve the imagery modal dataset alone, which includes the original high-resolution map images and their corresponding low-resolution map images. The dataset is available in the two aforementioned formats: .JPG, and .PNG.

The datasets of map style transferring also involves the imagery modal dataset. The dataset includes the original map images and the maps that shares the similar content but different styles. The dataset is available in the two aforementioned formats: .JPG, and .PNG.

## Technical Validation

### Validation of map sample collection

#### Readability and format

We used OPENCV, Adobe Photoshop, and ArcGIS to verify each collected map sample, ensuring that these files are machine-readable. For we invited these people with different education backgrounds so that diverse opinions could be considered in the labelling of each map. For map color and texture, noise, and inappropriate content, three individuals with different education backgrounds provided separate interpretation results. If at least one results were not same, we would make a further discussion on the results and determine the final results. Moreover, we would remove the map sample that no agreement could be given.

In addition, the benchmark dataset in the community of computer vision and pattern recognition typically provides the images in formats including.JPG and.PNG. Thus, we generate every map sample in these two formats so that machine intelligence techniques are capability of processing and addressing these map files.

#### Redundancy

Extending the diversity of one category is the critical criterion of benchmark dataset Thus, we employed the algorithms called structural similarity (SSIM) and normalized mutual Information (NMI) to measure the similarity among pre-processed maps shown in Fig. 3. The SSIM is expressed as follows,

2 $$ssim(i,j)=\frac{(2{m}_{i}{m}_{j}+{C}_{1})(2{\mathrm{cov}}_{i,j}+{C}_{2})}{({{m}_{i}}^{2}+{{m}_{j}}^{2}+{C}_{1})({{s}_{i}}^{2}+{{s}_{j}}^{2}+{C}_{2})}$$

where *i* and *j* are two maps, *m*_*i*_ and *m*_*j*_ refers to the mean of *i* and *j*, *s*_*i*_ and *s*_*j*_ refers to the variance of *i* and *j*, and cov_*x,y*_ refers to the covariance of *i* and *j*.

Moreover, the mean, variance and covariance of two maps are calculated based on their color space and grayscale.

The NMI is expressed as follows,

3 $$nmi(i,j)=2\frac{\sum _{i}\sum _{j}\,{p}_{i,j}\,\log \,\frac{{p}_{i,j}}{{p}_{i}{p}_{j}}}{{H}_{i}+{H}_{j}}$$

where *H*_*i*_ and *H*_*j*_ are the information entropy of two maps *i* and *i*, *p*_*i*_ and *p*_*j*_ refers to the marginal distribution of *i* and *j*, *p*_*i,j*_ refers to the joint distribution of *i* and *j*.

For the maps identified as similar by SSIM and NMI, we then manually checked these maps and removed the similar one.

### Map labelling validation

Map labeling validation was conducted in four categories: map scene labelling, map labelling for text annotation, and map labelling for super-resolution reconstruction. We designed a framework to evaluate the technical quality of each dataset by different approaches. A volunteer group involving two university faculties, four graduate students and two undergraduate students have joined the labelling validation. Since the labelling results might be varied based on different individuals, we invited these people with different education backgrounds so that diverse opinions could be considered in the labelling of each map. The details of validation are mentioned as follows.

#### Validation of map scene labelling

Five volunteers labelled the category of every map. If there were at least four label results were consistent, this map would be categorized under that label. Otherwise, we would discuss the labelled categories and decide which category was the appropriate. Moreover, if a map had no agreement among the volunteers, we would remove it from the CartoMark to avoid any confusions regarding map scene. Specifically, the removed maps would be stored for future using.

Moreover, we have used the labeled map scene datasets to conduct map type classification^20^ and multi-ask map type classification^21^.

#### Validation of map labelling for text annotation

In the first stage of checking, one volunteer labelled the text proposals of each map, and another volunteer checked the quality of the text proposal, which might vary in style, arrangement, and color, were accurately annotated. In the second stage of checking, the third volunteer would verify that the bounding box of each text proposal fully encompassed all text characters.

Moreover, we have used the labeled map text datasets to conduct map text annotation detection with transfer learning^25^.

#### Validation of map labelling for super-resolution reconstruction

To ensure the quality of low-resolution maps, we employed two commonly-used interpolation approaches: Gaussian image filtering and cubic spline interpolation to generate the low-resolution maps. The results generated by these two approaches have been used to evaluate the state-of-the-art machine intelligence techniques^29,30^.

Moreover, we have used the labeled map super-resolution datasets to conduct map super-resolution reconstruction^31^.

#### Validation of map style transferring labelling

Five volunteers checked the consistency among maps with different styles. If there were at least four label results were consistent, these maps would be used as the style transferring. Otherwise, we would discuss the consistency and decide whether these maps were appropriate. Moreover, if a map had no agreement among the volunteers, we would remove it from the CartoMark, and specifically stored them for future using.

### Program validation

All three programs were tested by different python encoding environments including Jupiter Anaconda, Pycharm, and Pysript. Moreover, these programs were tested on different operation systems including Windows (Windows 10 and Windows 11), Mac, and Ubuntu.

## Data Availability

All codes of programs mentioned in this manuscript could be found in the benchmark dataset, and are available to readers without undue qualifications.
